# Age-related changes in NG2-expressing telocytes of rat stomach

**DOI:** 10.1371/journal.pone.0249729

**Published:** 2021-04-06

**Authors:** Yasuhisa Tamura, Kumi Takata, Asami Eguchi, Mitsuyo Maeda, Yosky Kataoka

**Affiliations:** 1 Laboratory for Cellular Function Imaging, RIKEN Center for Biosystems Dynamics Research, Chuo-ku, Kobe, Japan; 2 Multi-Modal Microstructure Analysis Unit, RIKEN-JEOL Collaboration Center, Chuo-ku, Kobe, Japan; University of Minnesota Medical School, UNITED STATES

## Abstract

NG2 immunoreactive cells (NG2 cells) are found in the brain and peripheral tissues including the skin, intestinal tracts, and bladder. In a previous study, we observed the presence of NG2 cells in the stomach using bioluminescence imaging techniques in NG2-firefly luciferase (fLuc) transgenic (Tg) rats. Here, we aimed to identify and characterize NG2 cells in the adult rat stomach. Immunohistochemical studies showed that NG2 cells were mainly present in the lamina propria and most of the cells were gastric telocytes, co-expressing CD34, and platelet-derived growth factor receptor alpha (PDGFRα), with a small oval-shaped cell body and extremely long and thin cellular prolongations. In the rat stomach, NG2-expressing telocytes comprised two subpopulations: NG2+/CD34+/PDGFRα+ and NG2+/CD34+/PDGFRα-. Furthermore, we showed that the expression of NG2 gene in the aged rat stomach decreased relative to that of the young rat stomach and the decline of NG2 expression in aged rats was mainly observed in NG2+/CD34+/PDGFRα+ telocytes. These findings suggested age-related alterations in NG2+/CD34+/PDGFRα+ telocytes of rat stomach.

## Introduction

Telocytes were defined as a new type of interstitial cell in 2010 [1,2]. The cells are ultrastructurally characterized by a small cell body with a scarce cytoplasm and a variable number (one to five) of extremely long and thin prolongations, termed telopodes [3,4]. These cells are widely distributed in the tissues and organs of the body, including the heart, lungs, kidneys, liver and other tissues, even the skin [4,5]. Currently, it is believed that the most suitable markers for detecting telocytes are CD34 and platelet-derived growth factor receptor alpha (PDGFRα) [4,6]. It has been reported that telocytes have roles in intercellular signaling, immune and inflammatory responses, and tissue regeneration under physiological and pathological conditions [7–10].

NG2 immunopositive cells (NG2 cells) in the brain are known as oligodendrocyte progenitor cells and can undergo cell division and differentiate into oligodendrocytes and astrocytes, as well as neurons [11–18]. In addition, NG2 cells are also present in several peripheral tissues, including skin, small and large intestines, and kidney [19–22]. In terms of the gastrointestinal system, NG2 cells in the small and large intestines are myofibroblasts in the lamina propria and smooth muscle cells in the lamina muscularis mucosae and tunica muscularis of adult mice [19] and also are pericytes in the gut [23]. It has been known that alpha smooth muscle actin (αSMA) and PDGFRβ are known as markers for myofibroblasts or smooth muscle cells [19] and pericytes [24,25], respectively. In addition, we observed the presence of NG2 cells in the stomach of rats via bioluminescence imaging and gene expression studies in our previous study [22]. However, the identification and characterization of NG2 cells in the stomach and age-related alterations of the cells remain unclear. The aim of this study was therefore to examine the localization and characterization of NG2 cells in the rat stomach and to investigate changes of NG2 cells in aged rat stomach. Here, we show that the majority of NG2 cells were located in the lamina propria of the gastric mucosa and were telocytes, exhibiting colocalization of CD34 and PDGFRα. Furthermore, NG2-expressing telocytes were composed of two subtypes based on the expression of PDGFRα. Compared with young rats, the expression of NG2 declined in the aged rat stomach. Furthermore, the decrease of NG2 expression in the aged stomach was mainly observed in the NG2+/PDGFRα+ telocytes.

## Materials and methods

### Ethical approval

All animal experimental procedures were approved by the Ethical Committee on Animal Care and Use of RIKEN Center for Biosystems Dynamics Research (MA2009-17-20) (1 April 2018), and all experiments were performed in accordance with the Principles of Laboratory Animal Care (NIH publication No. 85–23, revised 1985).

### Animals

Normal Wistar rats were obtained from Japan SLC (Shizuoka, Japan) and were bred to establish NG2-firefly luciferase (fLuc) transgenic (Tg) rats, expressing the fLuc transgene in NG2 cells [22]. Young (2–3 months) and aged (22–24 months) Tg rats were housed under a 12/12 h light/dark cycle at 22 ± 2°C and 55% ± 10% humidity with free access to a standard diet and water.

Normal Wistar rats were deeply anesthetized using isoflurane and perfused transcardially with 0.01 M phosphate-buffered saline (PBS; pH 7.4). Then, the rodents were perfused and fixed with 4% formaldehyde (PFA) solution in 0.01 M PBS and 0.1% glutaraldehyde/4% PFA mixture in 0.01 M PBS for immunohistochemical and electron microscopy analyses, respectively.

Tg rats were employed in *ex vivo* bioluminescence imaging under anesthesia. After *ex vivo* bioluminescence imaging, stomach tissues isolated from all Tg rats were immersed in 4% PFA at 4°C for 2 h for histochemical studies or soaked in fresh RNAlater solution at 4 °C overnight for gene expression analysis.

### Immunofluorescence staining

Immunostaining studies were performed as described previously [22]. Stomach tissues were removed and post-fixed in a 4% formaldehyde solution in 0.01 M PBS at 4°C for 2 h, then soaked in 30% sucrose solution. Stomach sections (40 μm thick) were prepared using a cryostat microtome and incubated with monoclonal mouse anti-NG2 IgG (1:100, Abcam, Cambridge, MA, USA), polyclonal rabbit anti-NG2 IgG (1:100, Abcam), monoclonal rat anti-CD34 IgG (1:100, Santa Cruz, CA, USA), polyclonal rabbit anti-PDGFRα IgG (1:200, Abcam), polyclonal rabbit anti-PDGFRβ IgG (1:100, Santa Cruz), monoclonal mouse anti-CD31 IgG (1:100, BD Biosciences, New Jersey, USA), monoclonal mouse anti-αSMA IgG (1:100, Abcam), or polyclonal goat anti-luciferase (Luc) IgG (1:50, Promega, Madison, USA) antibodies at 4°C for 15–20 h. After washing for 30 min with 0.3% Triton-X100 in PBS (PBST), the sections were incubated with the appropriate Cy2, Cy3, or Cy5-conjugated secondary antibodies (1:200, Jackson ImmunoResearch Laboratories, West Grove, PA, USA) at 4°C for 2–4 h and washed with PBST for 30 min. The sections were mounted with Hoechst dye 33258 (Nacalai Tesque, Kyoto, Japan.) and observed using a confocal laser microscope (Digital Eclipse C1; Nikon, Tokyo, Japan).

### Immunoelectron microscopy

The isolated stomach tissues were soaked in a 0.1% glutaraldehyde/4% PFA mixture at 4°C overnight and then soaked in 30% sucrose solution. Tissue slices (50 μm thick) were cut using a vibratome and washed in 0.01 M PBS. After washing, the sections were incubated in polyclonal rabbit anti-NG2 IgG antibody (1:200) at 4°C overnight and were incubated in the biotinylated goat anti-rabbit IgG antibody at 4°C for 3–4 h. Immunoreactivity was visualized with 0.035% diaminobenzidine (Sigma, St. Louis, MO) and 0.028% hydrogen peroxide in 0.01 M PBS. The sections were washed in 0.01M PBS and post-fixed in 1% osmium solution. Then, the slices were dehydrated in graded ethanol (70, 80, 90, 99.5, and 100%), and embedded in epoxy resin. Thin (70 nm) sections were cut with an ultra-microtome and were mounted on pieces of silicon wafer. Then, the sections were stained with uranyl acetate, lead citrate, and observed using a JSM-7900F scanning electron microscope (JEOL, Tokyo, Japan).

### Bioluminescence imaging study

Bioluminescence imaging studies were performed as described previously [22]. In this study, in vivo bioluminescence imaging was performed with NG2-fLuc Tg rats under laparotomy conditions. Tg rats were anesthetized with pentobarbital (50 mg/kg) and were placed on a warm plate in the light-tight imaging chamber of the bioluminescent imaging system equipped with a CCD camera (IVIS kinetics, Caliper Life Sciences, Hopkinton, MA, USA). Then, the abdominal skin of the rats was incised and rats were administered D-luciferin at a dose of 30 mg/kg (Promega) intravenously. Bioluminescence images were acquired every minute for 10 min after luciferin injection. Bioluminescence signal intensities from each sample were quantified via the region of interest (ROI) analysis using Living Image analysis software (Xenogen, Alameda, CA, USA). The signal intensity in the ROI was expressed in photons/sec/cm^2^/sr. Under laparotomy conditions, in vivo bioluminescence imaging could not be used for the exact quantification of bioluminescence signals from the stomach, due to contaminating bioluminescent signals from surrounding tissues. In addition, time-intensity curves showed that bioluminescence signals from the stomach reached maximum values within one minute, then declined with time, and started to achieve steady-state emission 10 min after the intravenous injection of luciferin. Based on these data, *ex vivo* imaging studies were performed after 10 min of luciferin injection.

For *ex vivo* bioluminescence imaging, Tg rats were anesthetized with pentobarbital (50 mg/kg), intravenously injected with D-luciferin, and sacrificed 10 min after luciferin injection. Then, several tissues were immediately removed and placed on a heated stage in IVIS imaging system chamber. Bioluminescence images were captured after 20 min using the IVIS imaging system. Bioluminescence signal intensities from each region were quantified using ROI analysis.

### Gene expression analysis

Total RNA was extracted from several tissues using the ISOGEN Kit (NIPPON GENE, Tokyo, Japan). Total RNA (1 μg) was reverse-transcribed into cDNA using the PrimerScript RT reagent Kit with gDNA Eraser (Takara Bio, Otsu, Japan). Real-time PCR was performed using the SYBR Green PCR Kit (Toyobo, Osaka, Japan) with a Thermal Cycler Dice Real-Time System (TP-800; Takara Bio) according to the manufacturer’s instructions. The 2^−ΔΔCt^ method was used to analyze the relative changes in gene expression. qPCR was performed with the following sense and antisense primers: for NG2 sense, 5’-AGGTAAGCATGATGTCCAGGTG-3’ and antisense, 5’-CAGTTGTGAGTGGAATGGCTTG-3’; for ribosomal protein S18 (rps18) sense, 5’-CTTCCACAGGAGGCCTACAC-3’ and antisense, 5’-GATGGTGATCACACGCTCCA-3’. The expression of rps18 was used as the reference gene for normalization.

### Statistical analysis

Data from each animal were presented as the mean ± SEM. Statistical analyses were performed using GraphPad Prism software (v6.0; GraphPad Software Inc., San Diego, CA, USA). Comparisons of two groups were performed using a Mann-Whitney’s U test. The significance threshold was assumed to be P < 0.05.

## Results

### Localization and characterization of NG2 cells in the adult rat stomach

**Fig 1:**
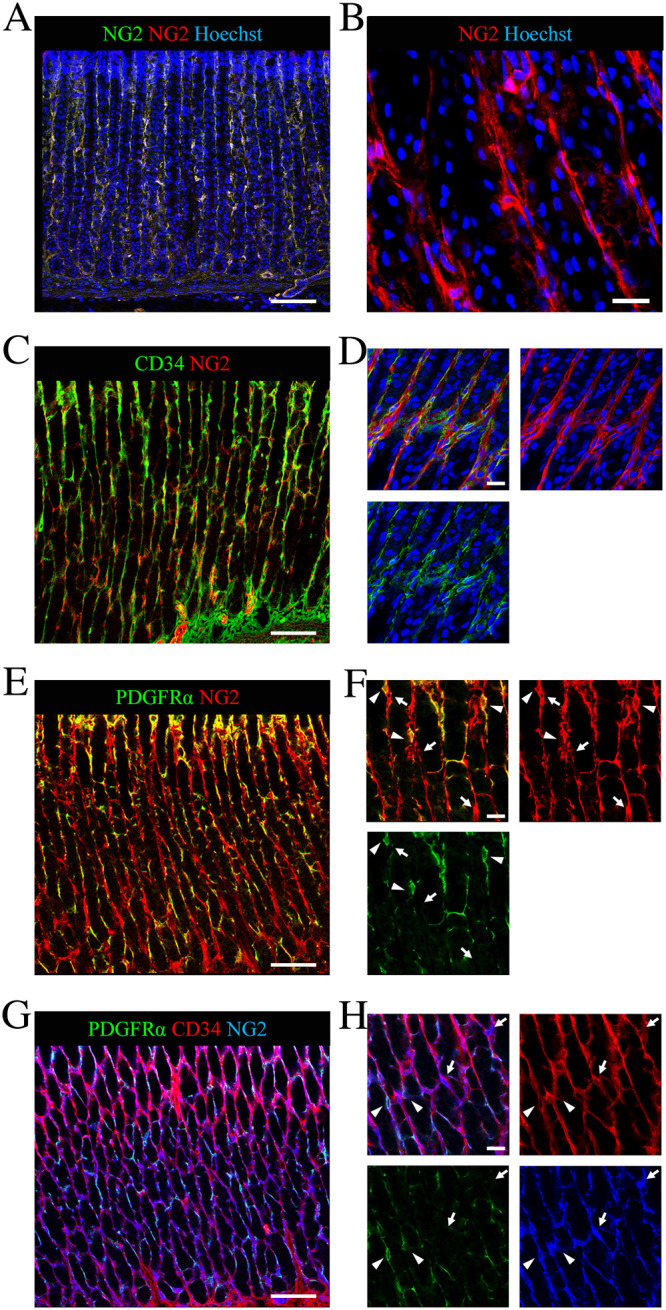
Localization and characterization of NG2 cells in the stomach of adult rats. (A-B) Localization of NG2 cells in the gastric mucosa. (A) Immunofluorescent images showing staining with both monoclonal NG2 (green) and polyclonal NG2 (red) antibodies and Hoechst (blue). (B) Magnified view of immunofluorescent images showing staining for NG2 (red) and Hoechst (blue). (C-D) Colocalization of CD34 (green) in NG2+ telocytes (red) of the adult stomach. Immunofluorescence staining for NG2 (red), Hoechst (blue) and CD34 (green) (a telocyte marker). (E-F) Co-expression of PDGFRα (green) in NG2+ telocytes (red) in the gastric mucosa. Immunofluorescence staining for NG2 (red) and PDGFRα (green) (another telocyte marker). Arrowheads show NG2+/PDGFRα+ telocytes whereas arrows point to NG2+/PDGFRα- telocytes. (G-H) Triple immunofluorescence staining for PDGFRα (green), CD34 (red) and NG2 (blue). Arrowheads indicate NG2+/CD34+/PDGFRα+ telocytes and arrows show NG2+/CD34+/PDGFRα- telocytes. Scale bars: 100 μm (A, C, E, G) and 20 μm (B, D, F, H).

In the stomach, NG2 cells were mainly found in the lamina propria of the gastric mucosa and were absent or very rarely observed in the muscularis mucosa (Fig 1A). Many of them appeared to have long prolongations reminiscent of telocytes (Fig 1B). Immunofluorescence staining using NG2 and PDGFRα and/or CD34 antibodies showed that immunoreactivity for CD34 was found in most of the NG2 cells (Fig 1C and S1A Fig), but was not observed in a few NG2 cells in the lamina propria (Fig 1D). Likewise, many NG2 cells were also immunoreactive for PDGFRα (Fig 1E), whereas some NG2 cells were not immunopositive for PDGFRα in the lamina propria (Fig 1F). To confirm the relationship between these cells, triple immunofluorescence staining was performed using NG2, PDFGRα, and CD34 antibodies. Images showed that both NG2+/CD34+/PDFGRα+ and NG2+/CD34+/PDFGRα- cells were present in the lamina propria, but NG2+/CD34-/PDFGRα+ cells were not found (Fig 1G and 1H). Based on these results, we divided the NG2+/CD34+ cells in the lamina propria into two subpopulations based on PDGFRα expression. In addition, to investigate the characterization of NG2+/CD34- cells (Fig 1D), immunostaining studies were performed using NG2 and αSMA or PDGFRβ antibodies. NG2 cells in the lamina propria and muscularis mucosae of gastric mucosa did not show the immunoreactivity for αSMA (S2A Fig). Meanwhile, the immunoreactivity for PDGFRβ was observed in some NG2 cells (S2B Fig). Moreover, to confirm whether PDGFRβ-expressing cells are pericytes based on their location (wrapped around endothelial cells), double immunostaining was performed with PDGFRβ and CD31 antibodies. Most PDGFRβ-expressing cells were located next to CD31+ endothelial cells (S2C Fig).

**Fig 2:**
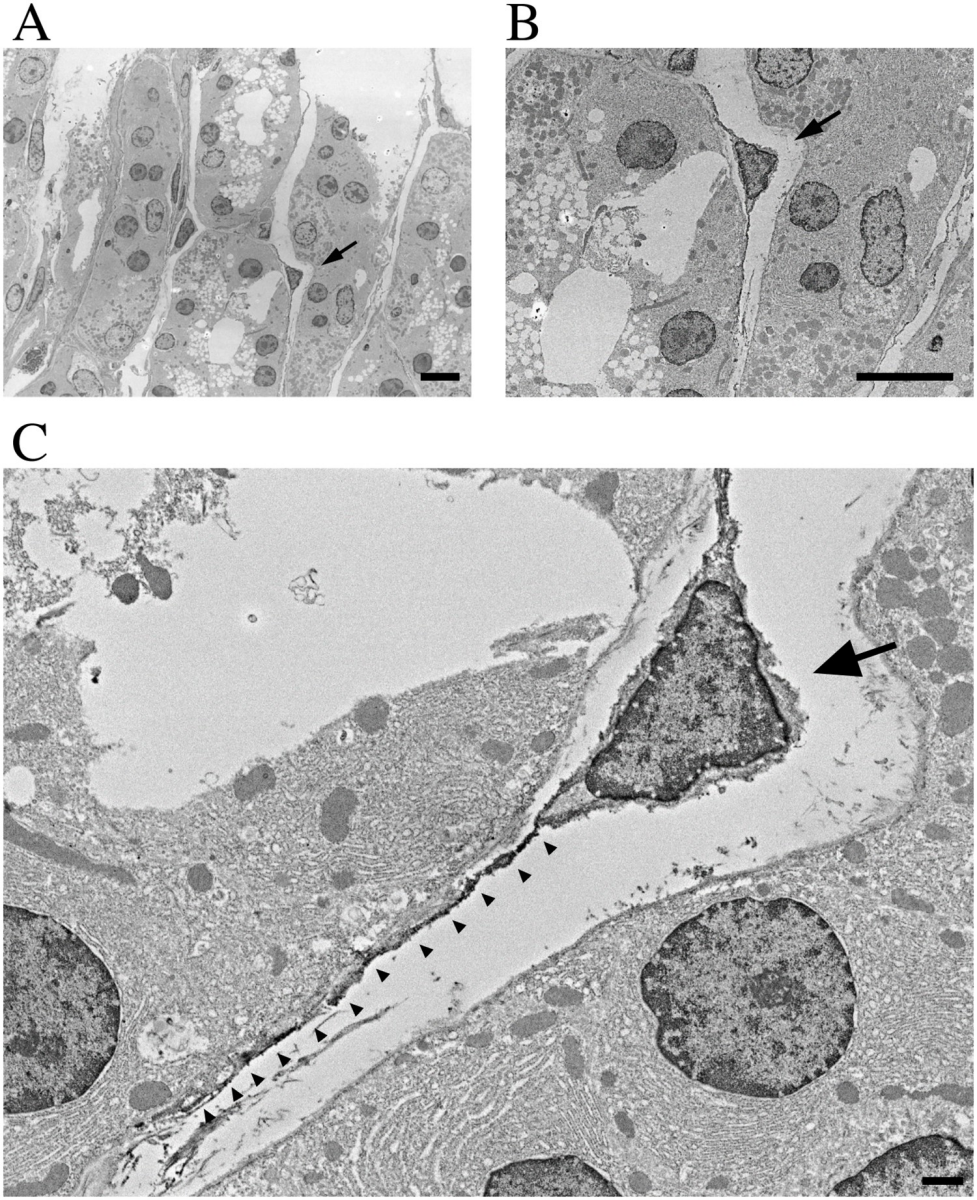
Identification of NG2+ telocytes in rat stomach using electron microscopy. (A-C) Representative electron micrograph of NG2-labeled gastric telocytes in the adult rat. (B-C) Magnified views of NG2 immunoreactive cell (arrow) in panel (A). (C) Arrowheads show cellular processes, stained with the anti-NG2 antibody. Scale bars: 10 μm (A-B) and 1 μm (C).

We confirmed that the NG2-expressing cells in the mucosa of the rat stomach were telocytes using immunoelectron microscopy with an antibody against NG2. The cells displaying NG2 immunoreactivity showed a small oval-shaped cellular body and two to three long prolongations in young rat stomach tissues (Fig 2). From these findings, we confirmed that NG2 cells in the adult rat stomach could be defined as gastric telocytes.

### Alterations in NG2 expression in the aged rat stomach at the tissue level

**Fig 3:**
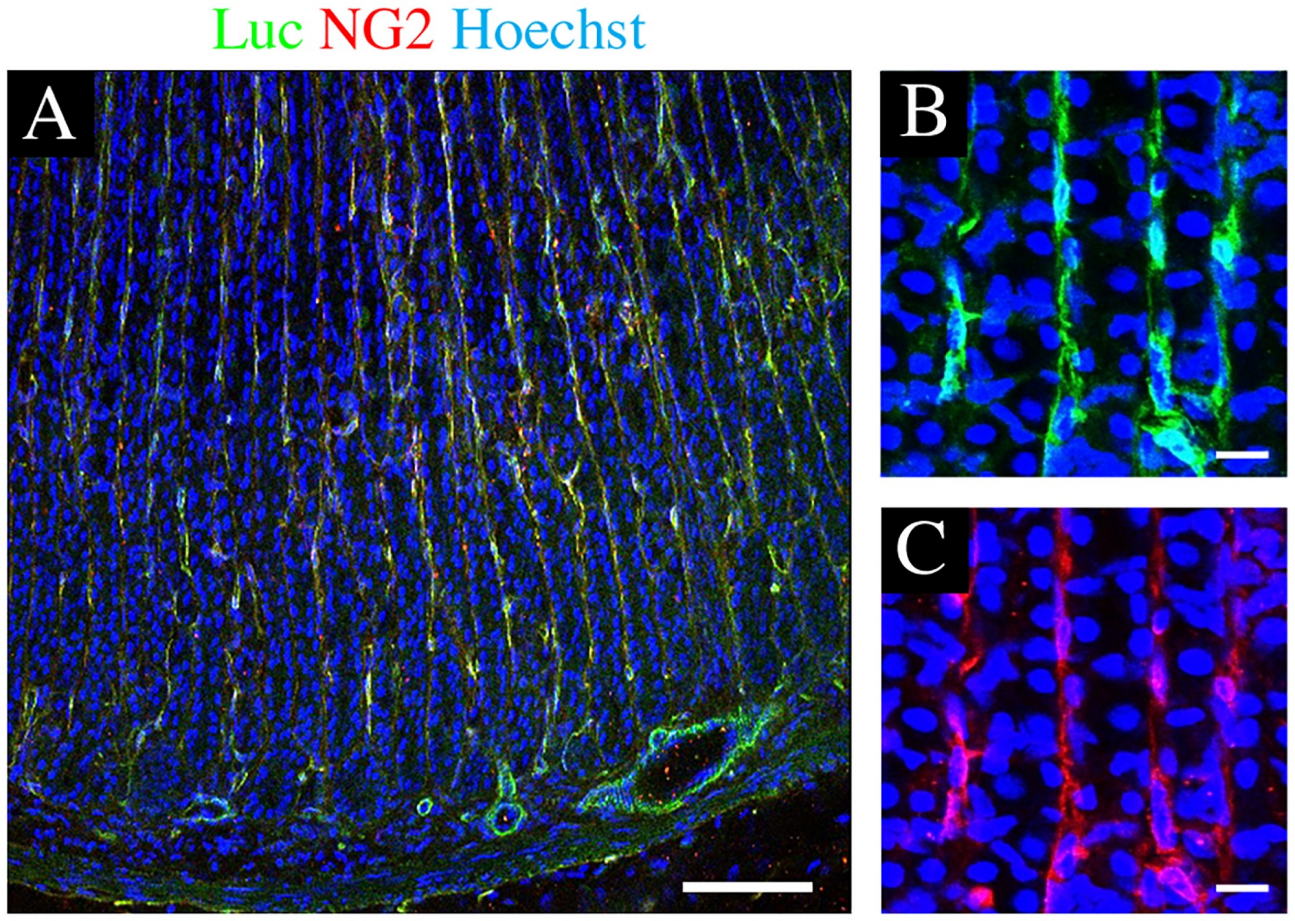
Expression of the fLuc reporter gene in NG2 cells of Tg rat stomach. (A-C) Co-expression of the fLuc transgene was observed in almost all gastric NG2+ cells of Tg rats. Immunofluorescence images of stomach sections stained with anti-NG2 (red) and anti-luciferase (green) antibodies and Hoechst (blue). Scale bars: 100 μm (A) and 20 μm (B-C).

**Fig 4:**
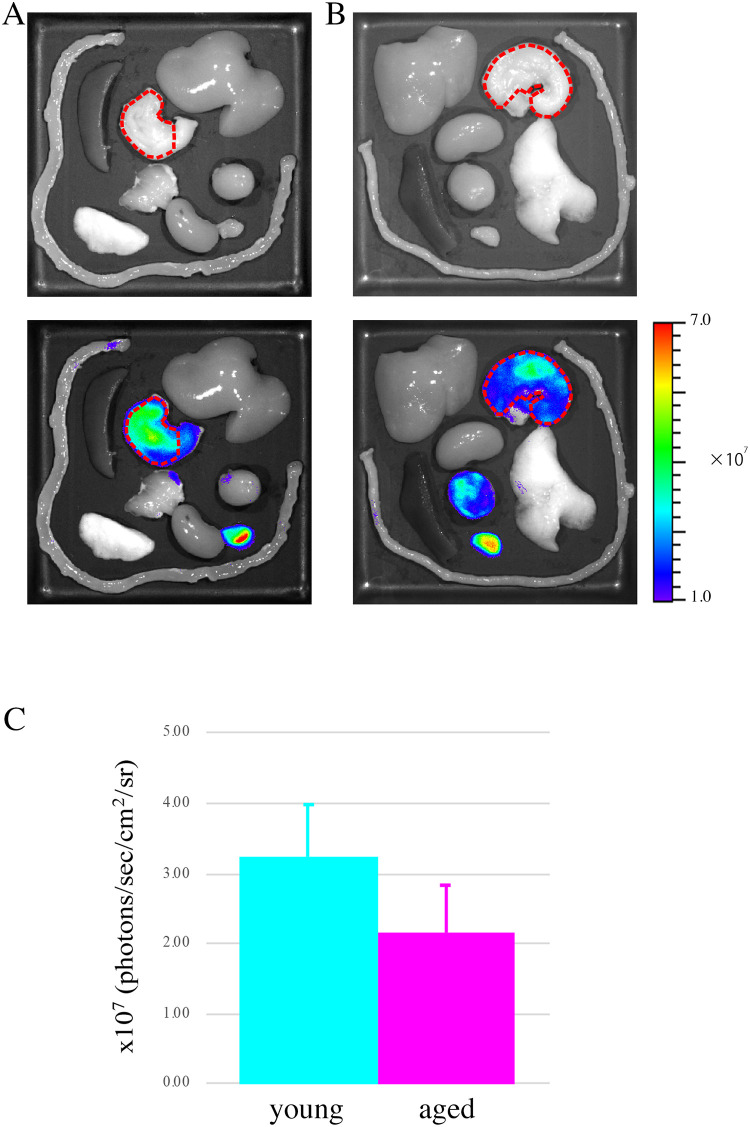
*Ex vivo* bioluminescence imaging of rNG2-fLuc Tg rats. (A-B) Bioluminescence images of several organs removed from young (A) and aged (B) Tg rats. The area indicated by red dashed lines includes the stomach and shows regions of interest (ROI). Upper panels: Photographs of several organs. Lower panels: Bioluminescence images of these organs. (C) Quantification of bioluminescence signals from the isolated stomach of young (light blue bar, n = 8) and aged (pink bar, n = 8) Tg rats.

**Fig 5:**
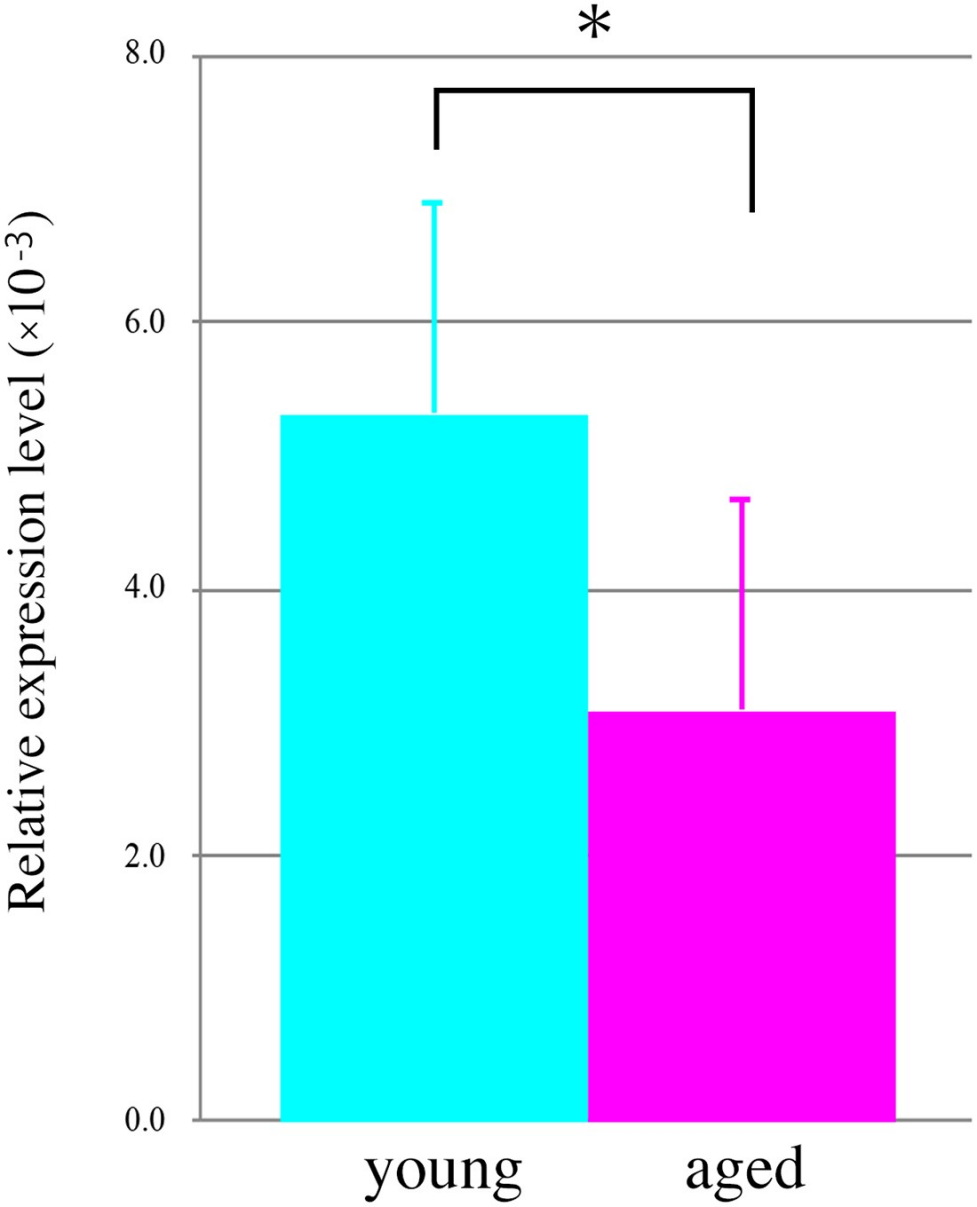
Comparison of NG2 gene expression in the stomach of young and aged Tg rats. NG2 expression in young (light blue bar, n = 6) and aged (pink bar, n = 6) stomach of Tg rats. Data are mean ± SEM. * p < 0.05.

First, we confirmed the expression of the fLuc transgene in gastric NG2 cells of the NG2-fLuc transgenic (Tg) rats via immunostaining prior to bioluminescence imaging. Almost all NG2-expressing cells also showed immunoreactivity for the fLuc transgene in the gastric lamina propria of the Tg rats (Fig 3). *Ex vivo* bioluminescence imaging was performed using both young and aged Tg rats. The bioluminescence signals from aged rat stomach tissues exhibited a decreasing trend compared to those from young rats (Fig 4A and 4B). Indeed, signal intensities from both young and aged stomach tissues were 3.25 ± 0.73 (n = 8) and 2.16 ± 0.67 × 10^7^ photons/sec/cm^2^/sr, respectively, (p = 0.054) (n = 8) (Fig 4C). We also investigated the expression of NG2 using quantitative polymerase chain reaction (qPCR) to validate the results of bioluminescence imaging. In the aged stomach, the mRNA expression of NG2 was significantly decreased compared to that in the young stomach (p = 0.035) (Fig 5). These findings suggested an age-related decrease in the expression of the NG2 gene in the rat stomach.

### Age-related changes in NG2-expressing telocytes in the rat stomach

**Fig 6:**
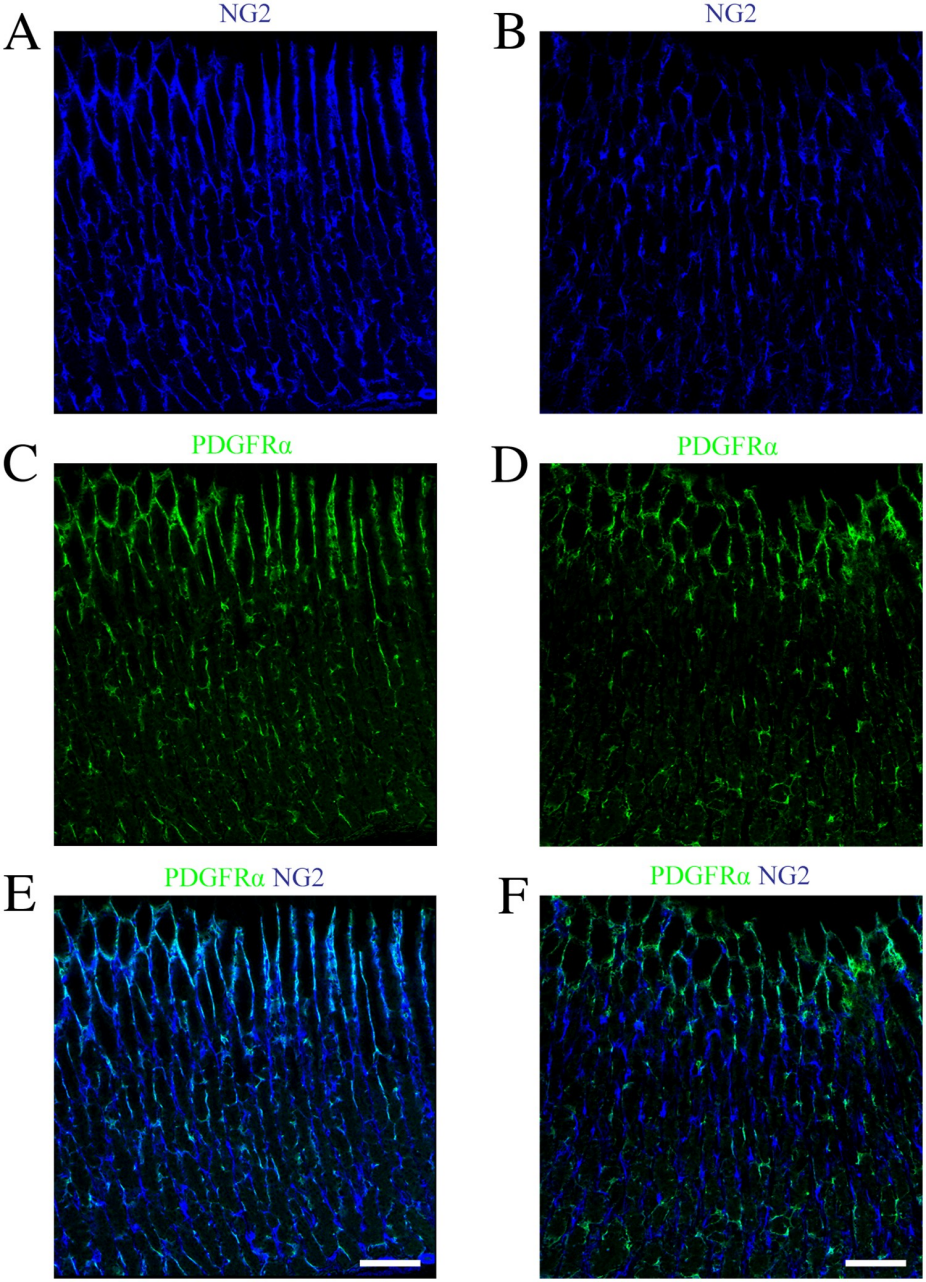
Age-related changes in NG2+ telocytes in the rat stomach. (A-F) Double immunofluorescence images of young (A, C, and E) and aged (B, D, and F) rat stomach sections stained with anti-NG2 (blue, A and B) and anti-PDGFRα (green, C and D) antibodies. (E, F) Merged images of NG2 (blue) and PDGFRα (green). Scale bar: 100 μm.

To date, age-related changes in gastric telocytes have not been described. At first, histological staining showed that interstitial spaces in the aged rat stomach tended to expand compared with those in the young rat stomach (S4 Fig). To examine the alteration of NG2-expressing telocytes in the stomach of aged rats, immunofluorescence staining was performed using an NG2 antibody. Immunoreactivity of NG2 in the aged stomach was lower than that in young rats (Fig 6A and 6B). We showed that gastric telocytes in the rats were composed of two subpopulations based on the expression of PDGFRα: NG2+/CD34+/PDGFRα+ and NG2+/CD34+/PDGFRα- (Fig 1). To investigate age-related changes of these cells, immunostaining studies were performed using an anti-PDGFRα antibody. The staining showed that the immunoreactivity of PDGFRα decreased in the aged stomach compared with that in the young stomach (Fig 6C and 6D). In addition, reduced immunoreactivity for NG2 was mainly found in NG2+/PDGFRα+ telocytes and was little or none in NG2+/PDGFRα- telocytes in the aged rat stomach. (Fig 6E and 6F).

## Discussion

In our previous study, we suggested that NG2 cells are present in the rat stomach using bioluminescence imaging [22]. However, the identification and characterization of these cells have not been clarified. Here, we showed that NG2 cells were mainly found in the lamina propria and were absent or rarely observed in the muscularis mucosae of the gastric mucosa in rats (Fig 1A and 1B). It has been reported that NG2 cells are present in both regions in the small intestine and colon [19]. Thus, the localization of NG2 cells in the stomach might be different from those in the mouse intestines. We divided the NG2 cells in adult rat stomach into two subpopulations according to the presence or absence of CD34 immunoreactivity (Fig 1D). In this study, NG2+/CD34+ cells were further separated into two subtypes with or without PDGFRα co-expression: NG2+/CD34+/PDGFRα+ cells and NG2+/CD34+/PDGFRα- cells (Fig 1E and 1H and S1B Fig). In mice intestine, PDGFRα-expressing cells consist of PDGFRα^high^ and PDGFRα^low^ cells based on the level of PDGFRα gene expression [26,27]. Moreover, PDGFRα^high^ cells are Forkhead box L1 (FoxL1)-expressing telocytes, whereas PDGFRα^low^ cells are composed of trophocytes or stromal cells based on their locations and functions in the lamina propria [26]. These findings suggest that NG2+/CD34+/PDGFRα+ cells and NG2+/CD34+/PDGFRα- cells in the rat stomach correspond to PDGFRα^high^ telocytes and PDGFRα^low^ stromal cells, respectively. Indeed, it was reported that gastric telocytes are CD34+/PDGFRα+ in humans [28] and Chinese giant salamanders [29]. However, the characterization of PDGFRα^low^ stromal cells remains to be elucidated. In the present study, both NG2+/CD34+/PDGFRα+ and NG2+/CD34+/PDGFRα- cells in the rat stomach showed typical morphological features of telocytes (S1B Fig). Future research is warranted to unveil these points. In contrast, NG2+/CD34- cells correspond to pericytes. Indeed, immunostaining studies showed that some NG2 cells were also immunoreactive for PDGFRβ, a marker of pericytes (S2B Fig) and that PDGFRβ+ cells were located near CD31-expressing endothelial cells (S2C Fig). Moreover, we showed that a small population of NG2 cells were also present adjacent to CD31+ endothelial cells, but NG2 and CD31 never co-localized in the adult rat stomach (S2D Fig). These data were confirmed in a previous study [23,30]. In a previous report, NG2 immunoreactivity was also observed in αSMA-expressing myofibroblasts and smooth muscle cells in the muscularis mucosa of mouse intestines [19]. However, NG2 cells in the adult rat stomach did not show αSMA immunoreactivity (S2A Fig). These data showed that NG2 cells in the gastric mucosa are composed of two subtypes of telocytes (NG2+/CD34+/PDGFRα+ and NG2+/CD34+/PDGFRα-) and NG2+/PDGFRβ+ pericytes. These observations are supported by a previous study [31]. At present, there are no reports describing the presence of telocyte subtypes in the stomach. However, subtypes of telocytes have been observed in the human urinary bladder [32] and human cornea [33]. Thus, these findings support our data that NG2-expressing telocytes consist of two subpopulations based on the absence or presence of PDGFRα expression.

To date, age-related changes of telocytes in the stomach remain to be fully elucidated. At the tissue level, the expression of NG2 in the aged (22–24 months old) rat stomach decreased compared with that in the young (2–3 months old) rat stomach, in both bioluminescence imaging and gene expression analyses (Figs 4 and 5). Correlation analysis showed that bioluminescence signals from the stomach were positively correlated with NG2 expression (correlation coefficient > 0.5) (S3 Fig) and that each dataset could be categorized according to age (S3 Fig).

Section staining studies showed that gastric interstitial spaces in the aged rat stomach appeared to enlarge compared with those in the young rat stomach (S4 Fig). These changes in the aged rat stomach have been reported [34]. At the cellular level, decreased expression of NG2 was observed in the lamina propria of the aged rat stomach compared to young rats (Fig 6A and 6B). The declined immunoreactivity for NG2 in the aged rat stomach was mainly found in NG2+/PDGFRα+ telocytes but was rare in NG2+/PDGFRα- telocytes (Fig 6C–6F). NG2 is a chondroitin sulfate proteoglycan and plays a role in cell migration and cell survival [35,36]. Therefore, the reduced expression of NG2 proteoglycan in NG2+/PDGFRα+ telocytes might be associated with the cell survival in aged rat stomach. Previous reports showed that structural changes in the perivascular regions are found in the aged rat stomach [34] and that reduced immunoreactivity for NG2 is observed in the pericytes in the small intestine of mice [37]. These findings indicated that age-related changes in the rat stomach might also be observed in perivascular cells, including pericytes.

In the present study, we used *ex vivo* bioluminescence imaging techniques to assess the cellular kinetics of NG2 cells in the young and aged rat stomach (Fig 4), because in vivo imaging methods cannot accurately quantify the signal intensities of NG2 cells in the stomach [22]. In preliminary studies, we attempted to perform in vivo bioluminescence imaging with Tg rats under laparotomy conditions. However, it was difficult to exclude surrounding tissues (including adipose tissue and other organs) from the stomach under these conditions, especially for an ROI analysis. A previous study reported the expression of NG2 in adipocytes [38]. Based on these findings, we performed *ex vivo* imaging studies to evaluate the cellular kinetics of stomach NG2 cells in both young and aged rats. Indeed, the results obtained from *ex vivo* imaging studies were supported by our gene expression data (S3 Fig), although we observed variations for each animal, especially in the aged group (S3 Fig).

Here we show that most NG2 cells in the rat stomach were gastric telocytes, expressing CD34 and PDGFRα and that NG2-expressing telocytes were composed of two subtypes based on PDGFRα expression. Expression of NG2 in the aged rat stomach decreased compared with that in young rats, revealed with bioluminescence imaging and gene expression studies. Furthermore, the decrease of NG2 expression in the aged stomach was mainly observed in NG2+/PDGFRα+ telocytes, but was not found in NG2+/PDGFRα- telocytes. These data suggest that age-related alterations are specifically observed in NG2+/CD34+/PDGFRα+ telocytes in the aged rat stomach.

## Supporting information

S1 Fig Colocalization of CD34 or PDGFRα in NG2+cells in the young rat stomach. (A) Z-stack images of immunofluorescence staining for CD34 (green) and NG2 (red). (B) Z-stack images of immunofluorescence staining for PDGFRα (green) and NG2 (blue). Arrowheads show NG2+/PDGFRα+ (red) and NG2+/PDGFRα- (white) cells with multiple cellular processes, whereas arrows point to NG2+/PDGFRα+ (red) and NG2+/PDGFRα- (white) cells, having long prolongations. Scale bars: 20 μm.
(PNG)

S2 Fig NG2 immunoreactivity in several interstitial cells of the young rat stomach. (A) Immunofluorescence staining for αSMA (green) and NG2 (red). (B) Immunofluorescence staining for PDGFRβ (green), NG2 (red), and Hoechst (blue). Arrowhead shows an NG2+/ PDGFRβ+ cell and arrows point to NG2+/ PDGFRβ- cells. (C) Immunofluorescence staining for PDGFRβ (green) and CD31 (red). (D) Immunofluorescence images of stomach sections stained with CD31 (green) and NG2 (red) antibodies. The arrow shows NG2+ cells adjacent to CD31+ endothelial cells. Scale bars: 20 μm.
(PNG)

S3 Fig Correlation between bioluminescence signal intensities and NG2 expression in young and aged Tg rats. Light blue diamonds: Young Tg rats (n = 6). Pink diamonds: Aged Tg rats (n = 6).
(PNG)

S4 Fig Comparison of tissue structures of young and aged rat stomach. (A-B) Images of young (A) and aged (B) stomach sections with hematoxylin and eosin staining. Scale bars: 100 μm.
(PNG)
