# Supplementary figures and images for: Age-related changes in NG2-expressing telocytes of rat stomach
Source: PLoS One. 2021 Apr 6;16(4):e0249729. doi: 10.1371/journal.pone.0249729 (PMC8023479; doi:10.1371/journal.pone.0249729)

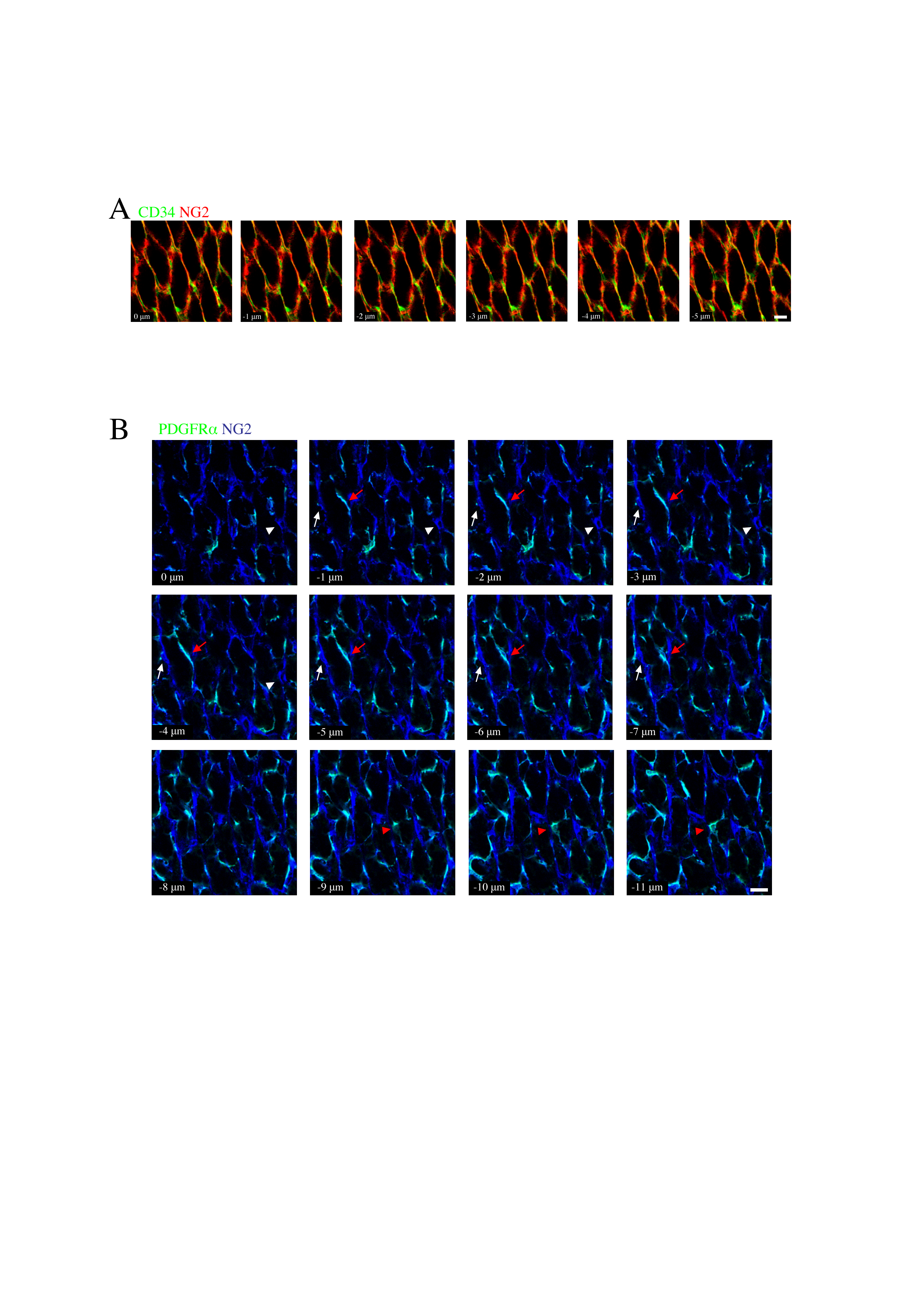

Supplement: S1 Fig — (A) Z-stack images of immunofluorescence staining for CD34 (green) and NG2 (red). (B) Z-stack images of immunofluorescence staining for PDGFRα (green) and NG2 (blue). Arrowheads show NG2+/PDGFRα+ (red) and NG2+/PDGFRα- (white) cells with multiple cellular processes, whereas arrows point to NG2+/PDGFRα+ (red) and NG2+/PDGFRα- (white) cells, having long prolongations. Scale bars: 20 μm. (PNG) [file pone.0249729.s001.png]

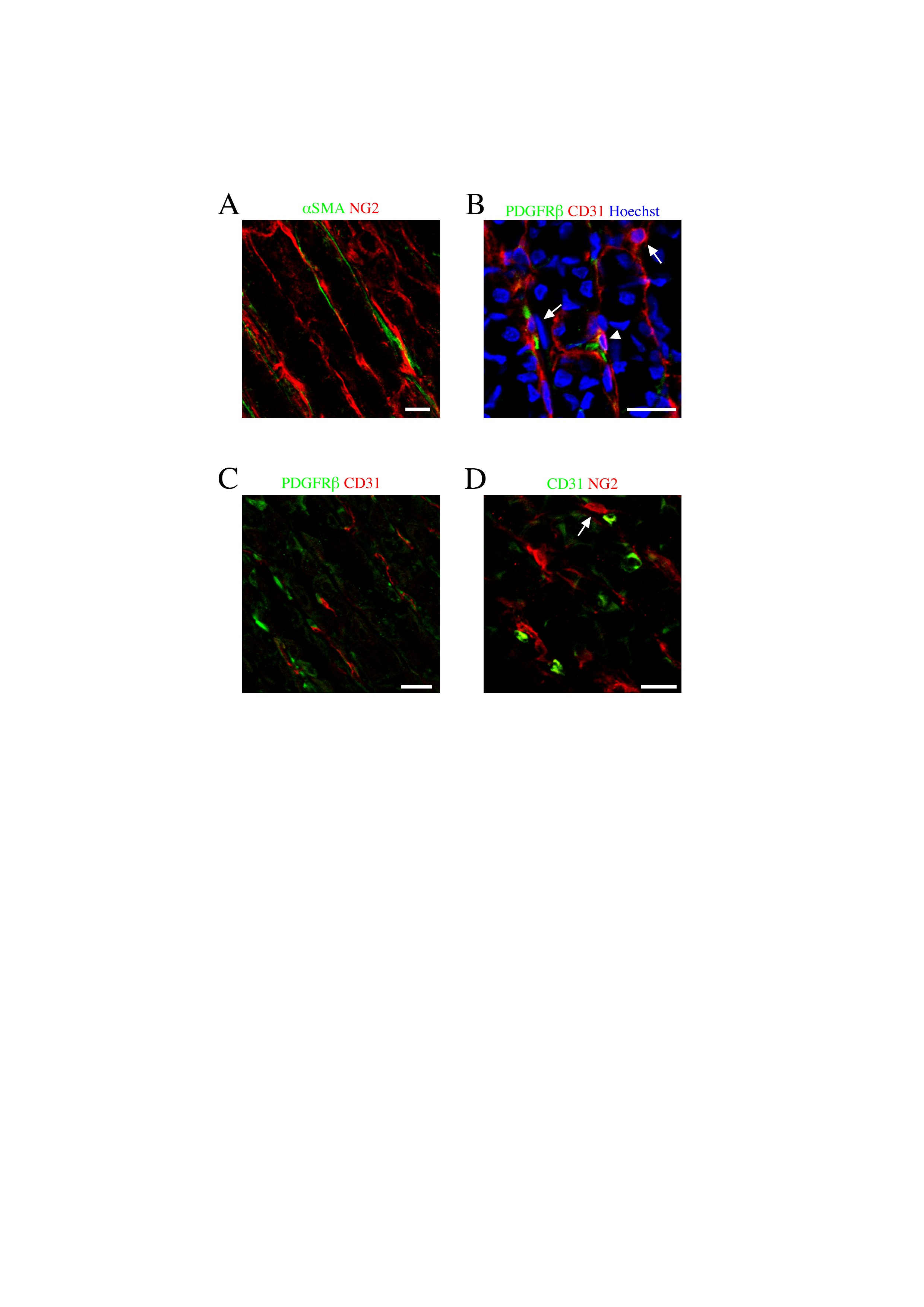

Supplement: S2 Fig — (A) Immunofluorescence staining for αSMA (green) and NG2 (red). (B) Immunofluorescence staining for PDGFRβ (green), NG2 (red), and Hoechst (blue). Arrowhead shows an NG2+/ PDGFRβ+ cell and arrows point to NG2+/ PDGFRβ- cells. (C) Immunofluorescence staining for PDGFRβ (green) and CD31 (red). (D) Immunofluorescence images of stomach sections stained with CD31 (green) and NG2 (red) antibodies. The arrow shows NG2+ cells adjacent to CD31+ endothelial cells. Scale bars: 20 μm. (PNG) [file pone.0249729.s002.png]

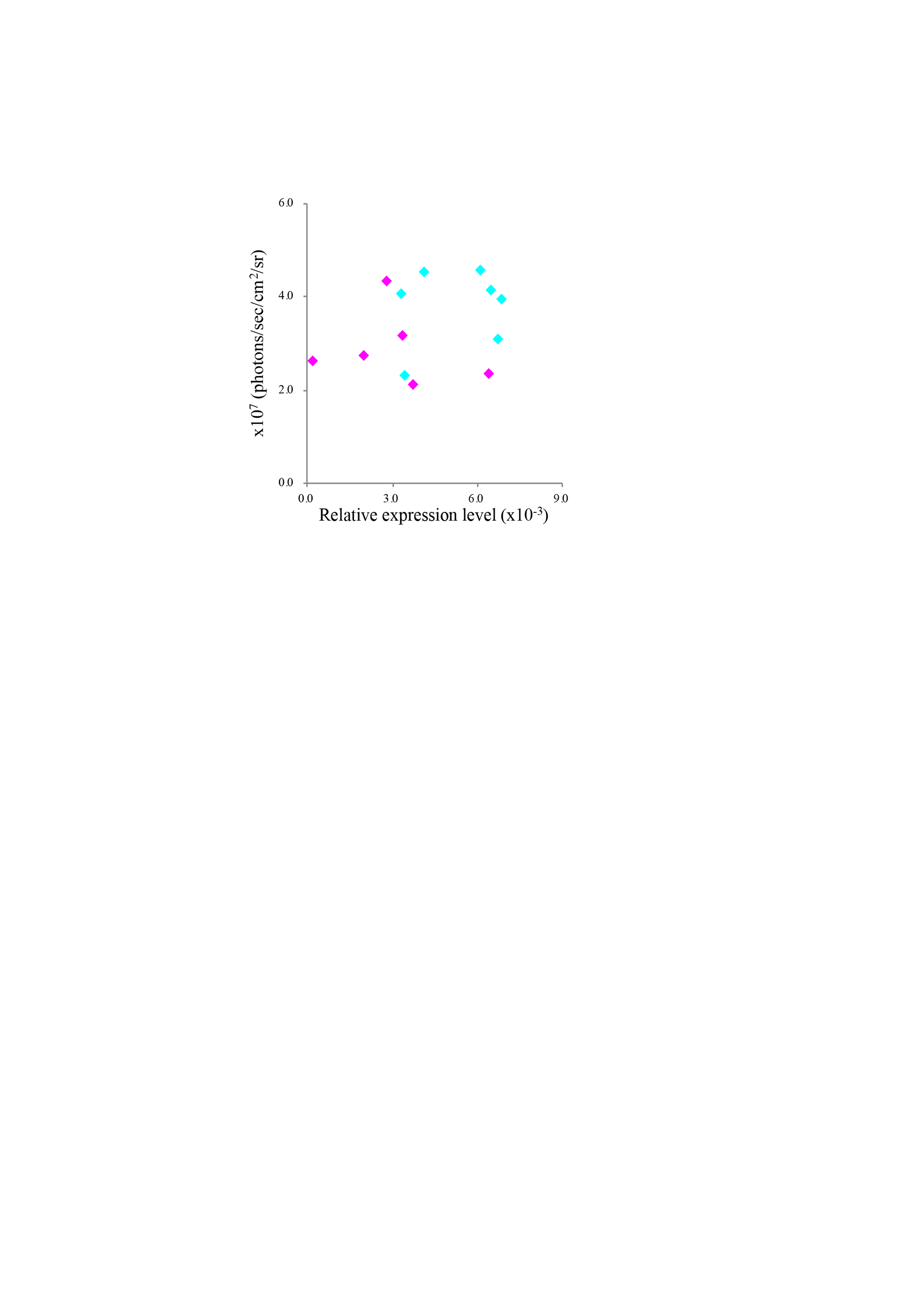

Supplement: S3 Fig — Light blue diamonds: Young Tg rats (n = 6). Pink diamonds: Aged Tg rats (n = 6). (PNG) [file pone.0249729.s003.png]

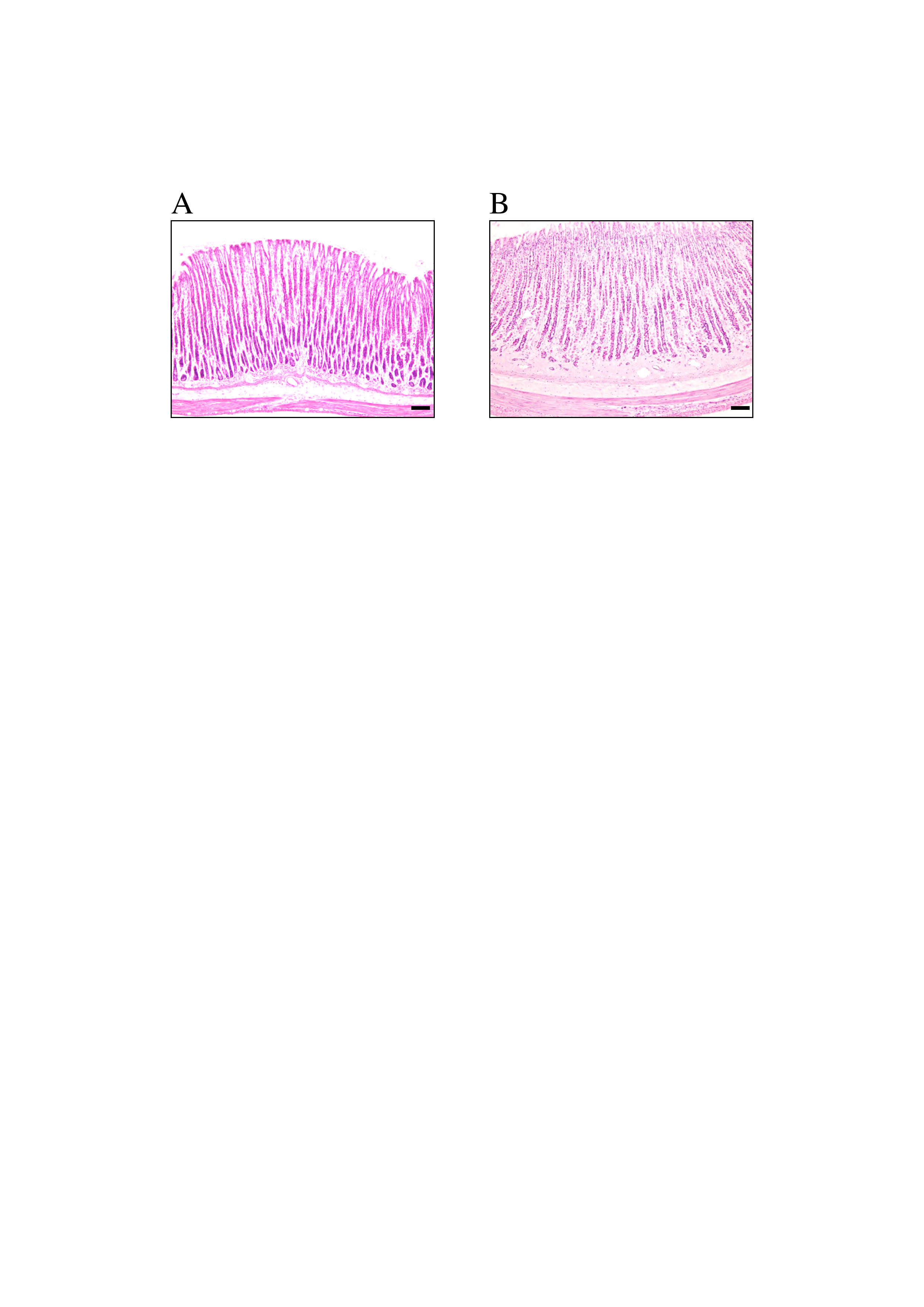

Supplement: S4 Fig — (A-B) Images of young (A) and aged (B) stomach sections with hematoxylin and eosin staining. Scale bars: 100 μm. (PNG) [file pone.0249729.s004.png]
